# Genetic diversity and environmental influence on morphological and yield parameters of maize in Benin

**DOI:** 10.1016/j.heliyon.2022.e09670

**Published:** 2022-06-09

**Authors:** Vincent Ezin, Chabrolle M.I. Kpanougo, Adam Ahanchede

**Affiliations:** Faculty of Agriculture, University of Abomey-Calavi, 01 BP 526, Cotonou, Benin

**Keywords:** Maize, Evaluation, Survey-collection, Earliness, Diversity

## Abstract

Seeds are the most important input in agricultural production and its quality seed determines the yield of the crop. They contribute to nearly 30% of crop productivity. The present study aims at surveying, collecting and characterizing maize accessions from the North, Centre and South Benin. Thirty-two (32) accessions were collected from 11 townships of Benin. Four experiments (Bassila, Glazoue, N'Dali and Adjohoun sites) were carried out to evaluate the agro-morphological characteristics of the collected 32 accessions. Eighteen (18) quantitative parameters were measured at each site. The accessions were classified into 3 groups by the hierarchical ascending classification showing a very important variability among cultivars with very highly significant (P < 0.001) or highly significant (P < 0.01) differences. The observed diversity among the different cultivars were based on plant height, growth life cycle, ear height, yield and yield components. The collection consisted of 18 intermediate-maturing cultivars showing big corn size with the ears inserted at a great height (Group 1), 8 late-maturing cultivars with the best vegetative and reproductive traits (Group 2) and 6 early-maturing cultivars with the best reproductive growth and best ear and grain yields (Group 3). The phenotypic variability of the cultivars was more related to their agronomic and morphological traits than their origin. These diverse groups of accessions could be used to develop improved maize varieties with high yield potential and adapted to different agro-climatic conditions.

## 1 Introduction

Maize is the staple crop largely cultivated in sub-Saharan Africa, grown in more than 33 million hectares per year [1]. They further indicated that it occupied approximately 17% of the estimated 200 million hectares of cultivated land in sub-Saharan Africa, and is grown in different environmental conditions.

Research on the best agronomic practices is of vital importance in optimizing yields and its components because of its role in food security [2, 3]. Indeed, it is widely used for human food and animal feed and serves as a raw material in some industries [4]. In the Beninese economy, agriculture remains the mainstay accounting for 70% of the working population and contributes nearly 33% to the GDP [5]. Maize cultivation, among cereals, is the most important in terms of production and contributes significantly to the income and social growth of the country [6, 7]. Maize occupies the first place in the national food system and remains the most consumed cereals ahead of rice and sorghum [8]. In the past ten years, despite the introduction of new maize production technologies such as improved high-yielding seed varieties, production in the country has not grown exponentially contrary to the wishes of the government. The yield has dropped from 1421 kg/ha to 1281 kg/ha in 2011 and 1304 kg/ha in 2018 while the area planted has increased from 82,016 ha in 2011 to 115,780 ha in 2018 [1].

Poor climatic conditions, non-availability of farm labour and low adoption of improved varieties are among the reasons that could explain this observed boom and bust development [9, 10]. Indeed, seeds are the most important input in agricultural production [11, 12]. It is then urgent to develop high yielding and resilient varieties to boost maize production. According to [13], local varieties represent essential plant material used by farmers and are the raw material for breeders to improve productivity and nutritional quality. They also present a better adaptation to the climatic and soil conditions of their region of origin [14].

To obtain newly developed cultivars of high quality in terms of productivity and stability, significant genetic diversity is key to the breeders. The *in situ* conservation of maize seeds by farmers, as well as their management, resulted in significant genetic diversity within cultivated maize varieties [15]. The use of this genetic diversity is of particular importance for maintaining and improving the productivity of this species in developing countries [16]. Numerous procedures exist to quantify and analyze genetic diversity. There are evaluation techniques using morphological markers [17]. The characterization of morphological descriptors reveals phenotypic diversity as observed and selected by local farmers, the main actors in the management of varietal diversity [18]. Thus, the objective of this study was to collect and characterize agro morphologically the diversity of maize cultivars in Benin.

## 2 Materials and methods

### 2.1 Prospecting and collection

✓ Method

**Figure 1:**
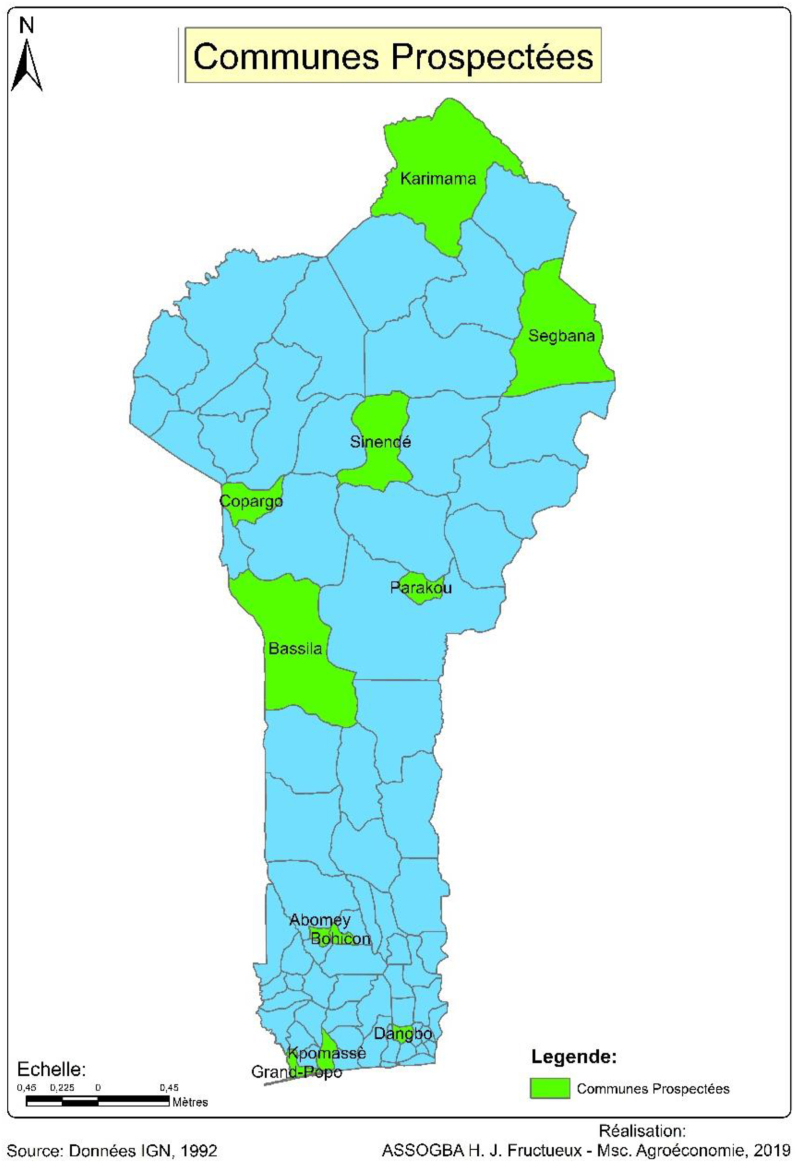
Map of surveyed communities.

A total of 32 maize cultivars were collected from eleven (11) township (two villages per township) in the North, Centre and South of Benin. These townships were Kpomasse, Grand popo, Dangbo, Abomey and Bohicon, Parakou and Sinende, Bassila and Copargo, Karimama and Segbana. Figure 1 shows in green the surveyed townships.

The choice of the surveyed villages was based on the guide of the presidents of the farmers’ associations in each township. The collection was therefore carried out in the most maize-producing villages. Maize ears were collected (at least five ears per cultivar) and preliminary information on the characteristics of each cultivar including length and width of ears, number of rows of grain per ear, colour of the seeds and number of seeds per row were performed. A GPS was used for the geographical coordinates of the survey areas.

### 2.2 Evaluation of agro-morphological characteristics of cultivars

✓ Study areas

Four environments were chosen for the experiments: one in southern Benin (Adjohoun site), one in the center part of the country (Glazoue), and two in the northern Benin (N'Dali in the Nord-Est and Bassila in the Nord-West). These areas are part of the major maize production zones of Benin and belong to different climatic zones: Adjohoun and Glazoue to the Guinean zone, Bassila to the Sudano-Guinean zone and N'Dali to the Sudanian zone.

✓ Adjohoun

The experimental site was located in the village of Goutin. The township of Adjohoun is characterized by a subtropical type of climate with two rainy seasons. The experiment was conducted from September 2018 to December 2018, thus during the small rainy season. The experimental soil was a ferralitic type soil was left fallow for 2 years. The previous crops grown on this soil were maize, cowpea and cassava. The vegetation was marked by the dominance of *Cyperus roduntus* and the presence of a few trees in some places such as *Oleais guinensis and Eucalptus*. The average rainfall during the trial was 79.75 mm.

✓ Glazoue

The experiment was conducted in the village of Akouegba from August to December 2018. The zone is characterized by a sub-equatorial climate, marked by two rainy seasons and two dry seasons. The soils were mainly of clay texture. The average rainfall during the experiment was 90.2 mm.

The vegetation was characterized by the presence of herbaceous plants with a dominance of Amaranthus. The crop history of the experiment plot was sorghum, cotton, and maize.

✓ Bassila

The experimental site was located in the village of Kantonneman. The experiment was conducted from August to December 2018. The climate is Sudano-Guinean type with only one rainy season (April to October) and a dry season (October to April). The average rainfall during the experiment was 105.8 mm.

The soil was of very old crystalline formations with a granite-gneissic mother rock favorable to crops. The vegetation was predominantly marked by herbaceous plants and some trees in some places such as *Khaya senegalensis*, *Azadirachta indica* and others. A short fallow of one year was observed on the plot before the experiment was conducted and the crop history was mainly maize.

✓ N'Dali

The experimental site was in the village of Suanin. The trial was carried out from August to December 2018. The site was characterized by a ferruginous soil. The climate is of the continental Sudano-Guinean type characterized by a rainy season (April to October) and a dry season (October to April) with the harmattan weather from November to February. The average rainfall during the trial was 106.8 mm. It is a very favourable climate for agriculture and livestock. There were herbaceous plants with some trees such as *Azadirachta indica*, *Mangifera indica*, and *Anacardium occidentale*. Millet and sorghum were cultivated in the plot before the experiment.

## 3 Method

### 3.1 Experimental design and crop management

The area of the experimental plot was 2000 m^2^ per site. The design was an alpha lattice design with three replications. The treatments were the 32 cultivars collected. Each replication was of eight blocks, each containing four treatments. Each treatment constituted experimental unit. Each experimental unit had an area of 6.4 m^2^ (4 m × 1.6 m). The distance between two experimental units was 0.8m, between two blocks 1m and between two replicates 2m. 25–30 cm deep ploughing was done followed by the making of 4m long ridges. The seeds were sown on the ridges with 10 hills per ridge at a rate of 2 seeds per hill. The distance between ridges was 0.80 m and 0.40 m between hills. Thus, each experimental unit was composed of three ridges. 25 kg of NPK were applied 15 days after sowing at a rate of 26 g per experimental unit and 0.86 g per plant. After the second weeding at 45 days after sowing, 25 kg of urea was applied to the plants.

A semi-selective herbicide treatment, Alligator 400 EC was applied to the experimental plot at a rate of 4 L per hectare, the following day after sowing. Weed control was then carried out manually using hoes. For the control of defoliating insects, Lambda Super 50EC was applied at the rate of 1 L per hectare as soon as the first attacks were observed.

### 3.2 Data collection from the field experiments

A total of 18 quantitative parameters were measured including morphological and agronomic traits using the maize descriptors [19]. These measurements were performed during the different maize development stages. Since maize is cross-pollinated crop, to avoid cross-fertilization and thus varietal mixture, self-fertilization was carried out on ten plants selected mainly on the middle row of each experimental unit. To this end, the tassels and silks of these plants were protected by envelopes. Self-pollination was then carried out by bringing pollen from the same plant onto the protected silks. After pollination, the silks were again covered with envelopes for four days.

### 3.3 Quantitative traits

✓ Morphological parameters:
  - Height of the plant (HPla)
  - Root collar diameter (CiCo)
  - Length (LonF) and width (LarF) of the leaf of the highest ear of the self-pollinated plants from each experimental unit
  - Number of leaves above the highest ear (NfEp)
  - Height of the ear (HEpi) in centimetres
✓ Agronomic parameters
  - Days to 50% silking (DaFf)
  - Days to tasseling (DaFm)
  - Tassel length (LonP) in centimeters,
  - Distance of Tassel branching (DrPa) in centimeters,
  - Number of primary branches of the tassel (NbpP),
  - Days to maturity (CsMa)
  - Number of ears per plant (NepP)
  - Length of the ear (LoEp)
  - Diameter of the ear (DiEp) in millimetres
  - Number of grain rows per ear (NrgE)
  - Average number of grains per row (NmGr)
  - And weight of 1000 grains (WG)

### 3.4 Data analysis

The collected data on agro morphological characteristics were analyzed using R software. The data from each site were subjected to an individual analysis of variance (ANOVA) to explore the differences and variability between cultivars for all quantitative traits measured and the significance of the measured variables. Then R software was used to determine the correlation coefficients and the significance of the correlation between the different quantitative variables measured and to perform correlation matrix. ANOVA, principal component analysis (PCA) and hierarchical ascending classification (HAC) were performed only on the quantitative data.

## 4 Results

### 4.1 Survey and seed collection

The geographical coordinates, origin and names of the cultivars collected are presented in Table 1. A total of 32 local and improved varieties were collected. Most of these cultivars were collected from r storage facilities.

**Table 1:** Accessions collected during the prospection.

Accession code	Place of origin	Local name	Geographical coordinates
G01	Dangbo/Klogbomey	Gregoir/Alaba	N 06°35′15.0″ E 002°32′41.1″
G02	Dangbo/Monotokpa	Semole	N 06°34′44.7″ E 002°35′09.7″
G03	Dangbo/Monotokpa	Rodja	N 06°35′11.5″ E 002°35′11.1″
G04	Dangbo/Monotokpa	Blafou	N 06°35′11.5″ E 002°35′11.1″
G05	Dangbo/Monotokpa	Amanssawe	N 06°35′11.5″ E 002°35′11.1″
G06	Bassila/Bakini	EVTD	N 09°0′9″ E 1°40′7″
G07	Grand-popo	DMR	N 06°19′31.4″ E 001°50′54.4″
G08	Kpomasse/Nonvignon	Party	N 06°26′13.5″ E 002°04′47.7″
G09	Kpomasse/Nonvignon	Gbade wewe	N 06°26′13.5″ E 002°04′47.7″
G10	Kpomasse/Nonvignon	Kpomasse	N 06°26′13.5″ E 002°04′47.7″
G11	Abomey/Asankanmey	Adjakouin	N 07°11′08″ E 01°59′17″
G12	Abomey/Asankanmey	Tikou	N 07°11′08″ E 01°59′17″
G13	Bohicon/sodohomey	Gbade vovo	N 07°10′26.7″ E 02°03′55.5″
G14	Bohicon/sodohomey	Gbade wewe 2	N 07°10′26.7″ E 02°03′55.5″
G15	Karimama center	Tchintchinga	N 12°04′00″ E 3°10′60″
G16	Karimama center	DMR white	N 12°04′00″ E 3°10′60″
G17	Karimama center	White corn	N 12°04′00″ E 3°10′60″
G18	Segbana/Toubou	Massepois	N 10°55′40″ E 3°41′40″
G19	Segbana/Toubou	Segbana	N 10°55′40″ E 3°41′40″
G20	Segbana/Toubou	Masse of 2 months	N 10°55′40″ E 3°41′40″
G21	Segbana/Toubou	Massetia (red)	N 10°55′40″ E 3°41′40″
G22	Sinende center	My friend	N 10°20′41″ E 2°22′45″
G23	Sinende/Niaro	Synee 2000	N 10°20′41″ E 2°22′45″
G24	Sinende/Niaro	Gbesouanou (yellow 3 months)	N 10°20′41″ E 2°22′45″
G25	Sinende/Niaro	Gbesouanou (yellow 4 months)	N 10°20′41″ E 2°22′45″
G26	Sinende/Danrigourou	Kekerekou (yellow 2 months)	N 10°20′41″ E 2°22′45″
G27	Parakou/Arafath	Wamla isotinatoussou (4 months)	N 09°21′0″ E 02°37′0″
G28	Parakou/Arafath	Wamla isotinatoussou (3 months)	N 09°21′0″ E 02°37′0″
G29	Parakou/Arafath	Wamla seme (yellow)	N 09°21′0″ E 02°37′0″
G30	Copargo/Pabegou	QPM	N 09°49′60″ E 01°32′57″
G31	Copargo/Pabegou	DMR 2	N 09°49′60″ E 01°32′57″
G32	Bassila/Bakini	TZPB	N 09°0′9″ E 1°40′7″

### 4.2 Qualitative traits

The frequency of occurrence of the different modalities is variable for the four characters (Table 2). Tassel color (ColP) and silk color (ColS) revealed three variables with the same frequencies. In fact, for all the cultivars, 18.75% of the tassels and silks had green colour; 28.13% were purple and 53.12% were green-purple. 50% cultivars had yellow seeds, 25% cultivars had white seeds, 18.75% had purple seeds and 6.25% showed red seeds.

**Table 2:** Frequency of qualitative characteristics.

	Variables	Efficient	Total	Frequency (%)
ColS	VeSo	6	32	18.75
	PoSo	9	32	28.13
	VePS	17	32	53.12
ColP	VePa	6	32	18.75
	PoPa	9	32	28.13
	VePP	17	32	53.12
ColG	BlGr	8	32	25
	JaGr	16	32	50
	RoGr	2	32	6.25
	PoGr	6	32	18.75

### 4.3 Quantitative variability between cultivars

The ANOVA expressed that the effect of the accession × site interaction was very highly significant (p < 0.001) for all the traits measured except for the length of the ear (LoEp) which had a highly significant effect (p < 0.01), while the number of grains per row (NmGr) and of the thousand grain weight (PmG) demonstrated significant effect (p < 0.05). The effect of the accession × site interaction could be attributed to the difference in environmental conditions from one site to another. Also, the effect of accessions was significant (p < 0.05) for the mean number of grains per row (NmGr), highly significant (p < 0.01) for the weight of a thousand grains (PmG) and very highly significant (p < 0.001) for the other measured characters (Table 3). The NmGr trait therefore showed little variability among cultivars.

**Table 3:** Significance of quantitative variables with combined ANOVA.

Variables	Accessions	Accessions∗Environment
CiCo	<2.2e-16 ∗∗∗	<2.2e-16 ∗∗∗
CsMa	<2.2e-16 ∗∗∗	<2.2e-16 ∗∗∗
DaFf	<2.2e-16 ∗∗∗	1.472e-07 ∗∗∗
DaFm	<2.2e-16 ∗∗∗	1.189e-06 ∗∗∗
DiEp	<2.2e-16 ∗∗∗	<2.2e-16 ∗∗∗
DrPa	<2.2e-16 ∗∗∗	<2.2e-16 ∗∗∗
HEpi	<2.2e-16 ∗∗∗	<2.2e-16 ∗∗∗
HPla	<2.2e-16 ∗∗∗	<2.2e-16 ∗∗∗
LarF	9.566e-07 ∗∗∗	1.279e-12 ∗∗∗
LoEp	6.711e-05 ∗∗∗	0.003784 ∗∗
LonF	<2.2e-16 ∗∗∗	<2.2e-16 ∗∗∗
LonP	<2.2e-16 ∗∗∗	<2.2e-16 ∗∗∗
NbpP	<2.2e-16 ∗∗∗	<2.2e-16 ∗∗∗
NepP	5.129e-14 ∗∗∗	<2.2e-16 ∗∗∗
NfEp	4.663e-15 ∗∗∗	<2.2e-16 ∗∗∗
NmGr	0.04247 ∗	0.03197 ∗
NrgE	1.666e-09 ∗∗∗	1.443e-15 ∗∗∗
PmG	0.003828 ∗∗	0.024424 ∗

Signif. codes: 0 '∗∗∗' 0.001 '∗∗' 0.01 '∗' 0.05 '.' 0.1 ' ' 1.

Tables 4, 5, 6, and 7 present the variability expressed by the traits studied at each experimental site. At N'Dali, Bassila, Glazoue and Adjohoun, the coefficients of variation ranged from 3.53 to 36.29; 5.55 to 36.82; 5.43 to 38.25 and3.77 to 37.84, respectively. Only the variables, days to 50% silking (DaFf), days to tasseling (DaFm), days to maturity (CsMa) and ear diameter (DiEp) had low values of variation coefficient at all locations (˂10%).

**Table 4:** Descriptive statistics of quantitative parameters at N'Dali.

N°	Variables	Mean	CV(%)	Min	Max
1	CiCo	6.01	18.80	4.70	6.96
2	LonF	73.24	18.40	52.43	90.56
3	LarF	8.62	18.44	7.32	10.10
4	DrPa	9.20	34.78	6.36	12.46
5	LonP	30.82	23.19	27.06	37.23
6	NbpP	15.43	36.29	11.26	19.60
7	NepP	1.16	32.75	1	1.70
8	HEpi	89.38	25.65	63.43	120.53
9	HPla	166.81	17.29	135.86	208.50
10	NfEp	6.55	37.86	5.26	10
11	DaFm	51.92	6.97	47	55.33
12	DaFf	57.52	5.68	52.66	60.33
13	CsMa	94.47	3.53	85.66	97
14	LoEp	136.73	64.33	93.61	220.52
15	DiEp	50.44	9.95	41.70	56.72
16	NrgE	13.21	16.65	11.40	16.66
17	NmGr	28.84	19.86	17.73	35.16
18	PmG	300.05	26.09	208.66	465.66

**Table 5:** Descriptive statistics of quantitative parameters at Bassila.

N°	Variables	Mean	CV(%)	Min	Max
1	CiCo	7.60	22.85	5.43	7.60
2	LonF	67.79	16.41	64.16	72.93
3	LarF	10.07	11.22	9.43	10.68
4	DrPa	9.30	35.16	6.60	12.03
5	LonP	30.82	23.19	25.50	35.33
6	NbpP	13.66	36.82	10.26	17.86
7	NepP	1.01	12.87	1	1.10
8	HEpi	85.71	19.79	73.80	98.73
9	HPla	126.22	16.93	112.13	142.33
10	NfEp	4.85	23.71	4.06	5.56
11	DaFm	53.19	6.46	48	56.33
12	DaFf	57.93	5.55	53	60.66
13	CsMa	89.45	5.89	79.33	95
14	LoEp	107.84	20.12	101.23	124.49
15	DiEp	45.19	11.39	41.79	48.55
16	NrgE	12.60	12.69	11.46	14
17	NmGr	23.58	18.06	18.20	28.80
18	PmG	176.56	22.82	137.66	237.66

**Table 6:** Descriptive statistics of quantitative parameters at Glazoue

N°	Variables	Mean	CV(%)	Min	Max
1	CiCo	6.14	23.61	5.13	7.76
2	LonF	61.48	18.18	48.90	70.10
3	LarF	7.92	38.25	6.71	10.68
4	DrPa	9.40	27.65	7.10	11.50
5	LonP	34.30	19.06	29.13	40.90
6	NbpP	11.21	30.41	8.90	14.70
7	NepP	1.07	26.16	1	1.36
8	HEpi	61.20	28.82	41.26	93.03
9	HPla	122.97	20.10	92.43	150.16
10	NfEp	5.39	33.58	4.83	7.30
11	DaFm	51.81	7.43	46.33	55
12	DaFf	56.73	6.38	53	60
13	CsMa	89.56	5.43	79.33	95
14	LoEp	111.13	21.96	92.51	128.24
15	DiEp	43	9.65	40.25	47.97
16	NrgE	11.69	14.45	10.60	12.86
17	NmGr	23.58	26.67	15.33	32.33
18	PmG	136.40	21.98	105.66	177.33

**Table 7:** Descriptive statistics of quantitative parameters at Adjohoun

N°	Variables	Mean	CV(%)	Min	Max
1	CiCo	6.08	30.42	3.86	7.83
2	LonF	77.32	14.65	62.46	87.06
3	LarF	9.85	21.42	8.66	10.90
4	DrPa	9.98	33.56	7.10	13.63
5	LonP	36.48	10.63	33.83	38.50
6	NbpP	11.89	37.84	7.80	17.63
7	NepP	1.03	17.47	1	1.16
8	HEpi	73.27	28.30	50.90	112.03
9	HPla	157.30	16.66	121.83	181.50
10	NfEp	5.03	42.94	4.16	7.06
11	DaFm	48.35	6.47	45.66	53
12	DaFf	51.04	6.38	48	55.66
13	CsMa	87.23	3.77	85	91.66
14	LoEp	124.96	17.54	102.90	147.42
15	DiEp	48.97	10.82	44.73	53.69
16	NrgE	12.59	14.21	11	14.53
17	NmGr	28.51	23.99	21.44	38.66
18	PmG	247.07	13.80	226.66	278.33

### 4.4 Correlation between variables

The correlation matrix (Tables 8 and 9) indicates the types of relationships that exist between the different quantitative variables measured. The positive correlations obtained between root collar (CiCo) and plant height (HPla) (26.5%), between plant height and tassel branching distance (DrPa) (22.1%), between plant height and ear height (HEpi) (55.6%), between plant height and leaf length (LonF) (52,4%), between plant height and number of leaves above the ear (NfEp) (4.9%) revealed that plant height had a positive influence on root collar, tassel branching distance, ear height, leaf length and number of leaves above the ear. Thus, cultivars with great heights had a large root collar, long tassel branching, great height of ear, long leaves and medium number of leaves above the ear.

**Table 8:** Pearson correlation matrix between quantitative variables.

	CiCo	CsMa	DaFf	DaFm	DiEp	DrPa	HEpi	HPla	LarF	LoEp
CiCo	1.000									
CsMa	0.062	1.000								
DaFf	-0.008	0.297	1.000							
DaFm	0.010	0.212	0.945	1.000						
DiEp	0.054	0.078	-0.065	-0.031	1.000					
DrPa	0.041	0.046	0.052	0.075	0.078	1.000				
HEpi	0.171	0.195	0.228	0.228	0.208	0.313	1.000			
HPla	0.265	0.154	-0.057	-0.010	0.350	0.221	0.556	1.000		
LarF	0.069	-0.150	-0.111	-0.001	0.167	0.124	0.169	0.213	1.000	
LoEp	0.058	0.095	-0.133	-0.124	0.446∗	0.135	0.182	0.253	0.022	1.000
LonF	0.150	0.061	-0.073	0.005	0.277	0.248	0.393	0.524	0.380	0.199
LonP	0.068	-0.097	-0.138	-0.062	0.134	0.151	0.041	0.228	0.168	0.140
NbpP	0.135	0.177	0.147	0.101	0.102	0.412	0.459	0.330	0.018	0.168
NepP	0.061	0.079	0.069	0.056	0.027	0.142	0.174	0.128	-0.014	0.076∗
NfEp	0.038	0.114	0.057	0.025	0.050	0.043	0.026	0.049	-0.083	0.165
NmGr	0.099	0.092	-0.055	-0.010	0.510∗	0.136	0.189	0.358	0.171	0.416∗
NrgE	0.015	0.034	0.052	0.070	0.338∗	0.102	0.192	0.240	0.186	0.128∗
PmG	-0.031	0.239	-0.105	-0.097	0.527∗	0.079	0.272	0.454	0.095	0.439∗

CiCo = Root Collar, CsMa = days to maturity, DaFf = days to silking, DaFm = days to tasseling, DiEp = Diameter of ear, DrPa = Branching distance of ear, HEpi = Height of ear, HPla = Plant height, LarF = Leaf width, LoEp = Length of ear, LonF = Leaf length, LonP = ear length, NbpP = Number of primary branches of ear, NepP = Number of ear per plant, NfEp = Number of leaves above ear, NmGr = Average number of grains per row, NrgE = Number of rows of grains per ear, PmG = Thousand-grain weight, ∗ = significance.

**Table 9:** Pearson correlation matrix between quantitative variables (continued).

	LonF	LonP	NbpP	NepP	NfEp	NmGr	NrgE	PmG
LonF	1.000							
LonP	0.240	1.000						
NbpP	0.230	0.053	1.000					
NepP	0.107	-0.019	0.096	1.000				
NfEp	0.014	-0.036	0.038	0.151	1.000			
NmGr	0.344	0.162	0.190	0.076	0.044	1.000		
NrgE	0.217	0.049	0.134	0.061	-0.043	0.216∗	1.000	
PmG	0.304	0.096	0.273	-0.055	0.070	0.450∗	0.179∗	1.000

LonF = Leaf length, LonP = tassel length, NbpP = Number of primary branches of tassel, NepP = Number of ear per plant, NfEp = Number of leaves above ear, NmGr = Average number of grains per row, NrgE = Number of rows of grains per ear, PmG = Thousand grain weight, ∗ = Significance.

Also the weak correlations obtained between tassel length (LonP) and ear diameter (DiEp) (13.4%), between tassel length and tassel branching distance (DrPa) (15.1%), between tassel length and ear length (LoEp) (14%) between tassel length and number of primary tassel branches (NbpP) (5.3%) indicate that tassel length positively influences ear size, tassel branching, ear length and number of primary tassel branches. Thus, cultivars with long tassel had large and long ear, high tassel branching and high number of primary tassel branches.

A positive correlation was also observed among the yield parameters: number of ear per plant (NepP) and ear diameter (DiEp) (2.4%), ear length (LoEp) (7.6%), average number of grains per row (NmGr) (7.6%) and number of rows of grain per ear (NrgE) (6.1%). Therefore, the number of ear positively influenced the other yield parameters except for the thousand kernel weight (MGW) which showed a negative correlation with the number of ear. Thus, the number of ears per plant had a negative influence on the weight of these ears.

### 4.5 Principal component analysis (PCA)

Table 10 is the eigenvalues and cumulative percentages of the quantitative parameters on the axes. Five components with an eigenvalue greater than 1 were obtained, accounting for 73.14% of the variance present in the variables. The proportions of these eigenvalues, representing in fact the variances of the components, revealed that 26.98% of the initial information is explained by the first component, 47.41% by the second component, 58.79% by the third component and 66.18% by the fourth component. However, the variance accumulation test showed that the first four components were the most relevant. These four components were used to describe the total variability of the cultivars, which is 66.18% of the variance. They are therefore important to summarize most of the information related to the agro-morphological characteristics of these cultivars.

**Table 10:** Eigen value correlation matrix.

Component	Eigen value	Percentage of variance	Cumulative percentage of variance
**1**	4.856	26.980	26.980
**2**	3.677	20.429	47.409
**3**	2.048	11.378	58.787
**4**	1.330	7.393	66.180
**5**	1.252	6.958	73.138

**Figure 2:**
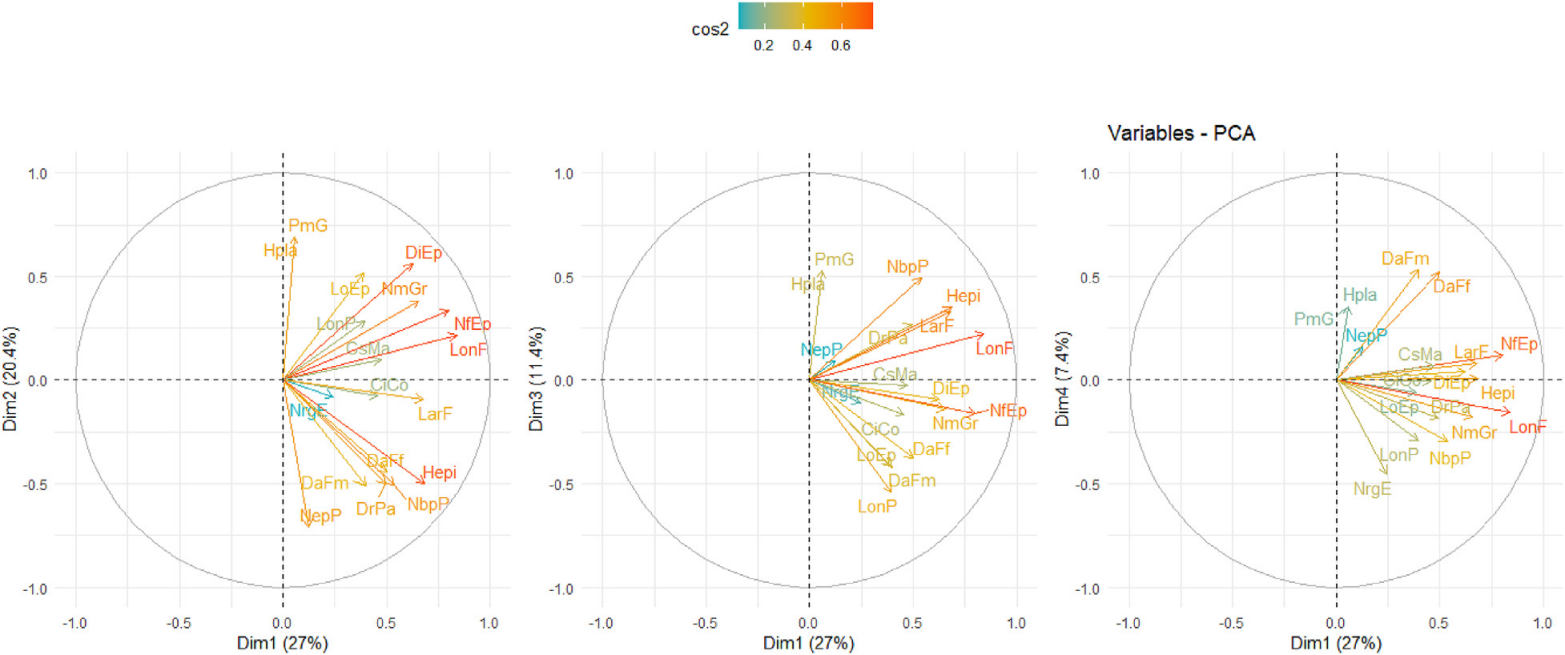
Correlation circle of morphological and agronomic traits per axis.

The four components were considered as axes and all variables are represented on these axes (Table 11, Figure 2). The projection of quantitative traits on the axis system 1, 2, 3 and 4 (Figure 7), revealed 3 distinct groups of traits.

**Table 11:** Contribution of quantitative variables to the formation of the first five axes.

N°	Variables	Axis 1	Axis 2	Axis 3	Axis 4	Axis 5
1	NrgE	0.245	-0.079	-0.111	-0.455	0.409
2	Hpla	0.057	**0.694**	0.53	0.353	0.184
3	NepP	0.123	**-0.71**	0.094	0.16	-0.036
4	LoEp	0.387	**0.514**	-0.417	-0.058	-0.116
5	DiEp	**0.625**	0.561	-0.091	0.043	-0.117
6	NmGr	**0.656**	0.384	-0.14	-0.177	0.263
7	PmG	0.057	**0.694**	0.53	0.353	0.184
8	CiCo	0.457	-0.076	-0.165	-0.002	**-0.648**
9	LonF	**0.839**	0.22	0.224	-0.157	-0.096
10	LarF	**0.679**	-0.092	0.329	0.079	-0.371
11	DaFf	0.5	-0.446	-0.377	**0.52**	0.188
12	DaFm	0.399	-0.508	-0.418	**0.532**	0.19
13	LonP	0.395	0.284	**-0.541**	-0.294	0.088
14	DrPa	0.494	**-0.501**	0.271	-0.183	0.427
15	NbpP	**0.538**	-0.507	0.495	-0.299	-0.024
16	NfEp	**0.801**	0.335	-0.162	0.122	-0.016
17	Hepi	**0.684**	-0.499	0.351	0.006	-0.098
18	CsMa	0.472	0.096	-0.024	0.071	0.284

The bold numbers mean the strong correlation between axes.

**Figure 3:**
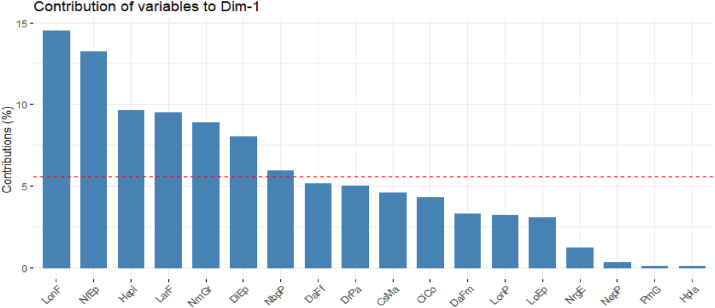
Contribution of the variables to the formation of axis 1.

**Axis 1** (26.98%): is defined by seven strongly positive correlations including seven variables namely: ear diameter (DiEp; 62.50%), average number of grains per row (NmGr; 65.60%), leaf length (LonF; 83.90%), leaf width (LarF; 67.90%), number of primary tassel branches (NbpP; 53.80%), number of leaves above the ear (NfEp; 80.10%) and ear height (HEpi; 68.40%). Axis 1 is the axis of cultivars with broad and long leaves, ear inserted very high on the plant, large and full of grains. It is therefore the axis of cultivars with the best vegetative development and best grain development (Table 11, Figure 3).

**Figure 4:**
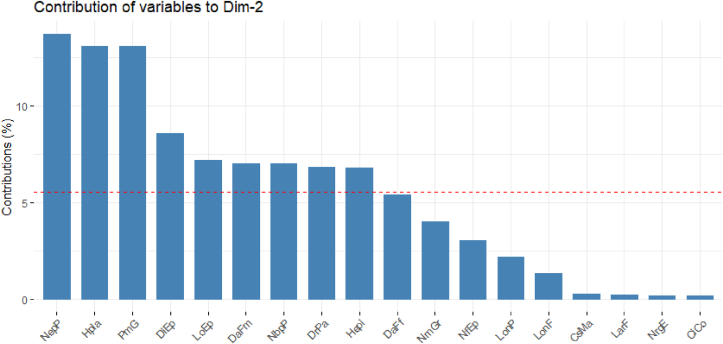
Contribution of the variables to the formation of axis 2.

**Axis 2** (20.43%): is defined by five variables, three of which are strongly positively correlated: plant height (HPla; 69.40%), ear length (LoEp; 51.40%) and thousand grain weight (PmG; 69.40%). On the other hand, this axis was negatively correlated with the number of ear per plant (NepP; -71.01%) and the distance between tassel branches (DrPa; -50.10%). It is therefore the axis of size and productivity (Table 11, Figure 4).

**Figure 5:**
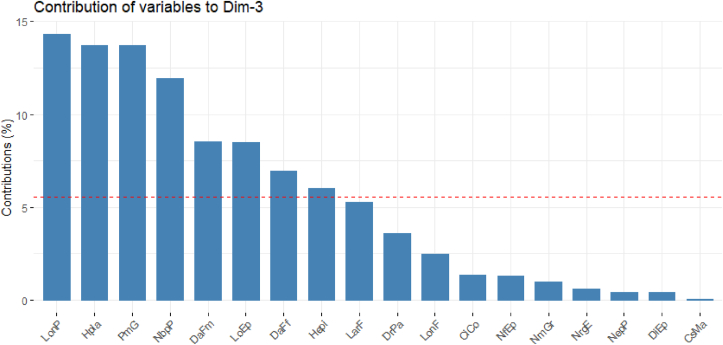
Contribution of the variables to the formation of axis 3.

Only one character contributed significantly to the formation of **axis 3**: tassel length (LonP; -54.10%) which is negatively correlated on this axis. This axis accounted for 11.38% of the total variability. It is the axis of cultivars with small tassel (Table 11, Figure 5).

**Figure 6:**
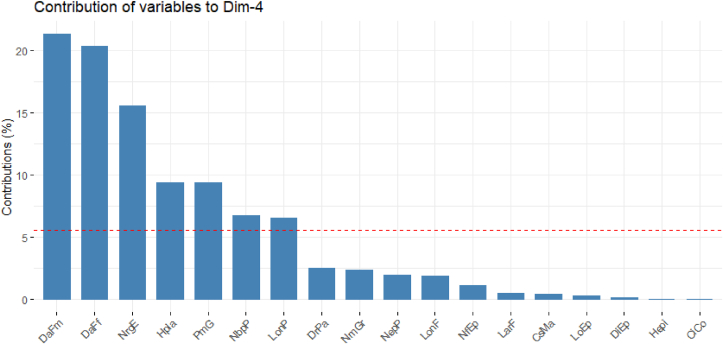
Contribution of the variables to the formation of axis 4.

**Axis 4** describes 7.39% of the variation. It is defined by days to silking (DaFf; 52%) and the days to tasseling (DaFm; 53.20%). It is the axis of late maturing cultivars (Table 11, Figure 6).

**Figure 7:**
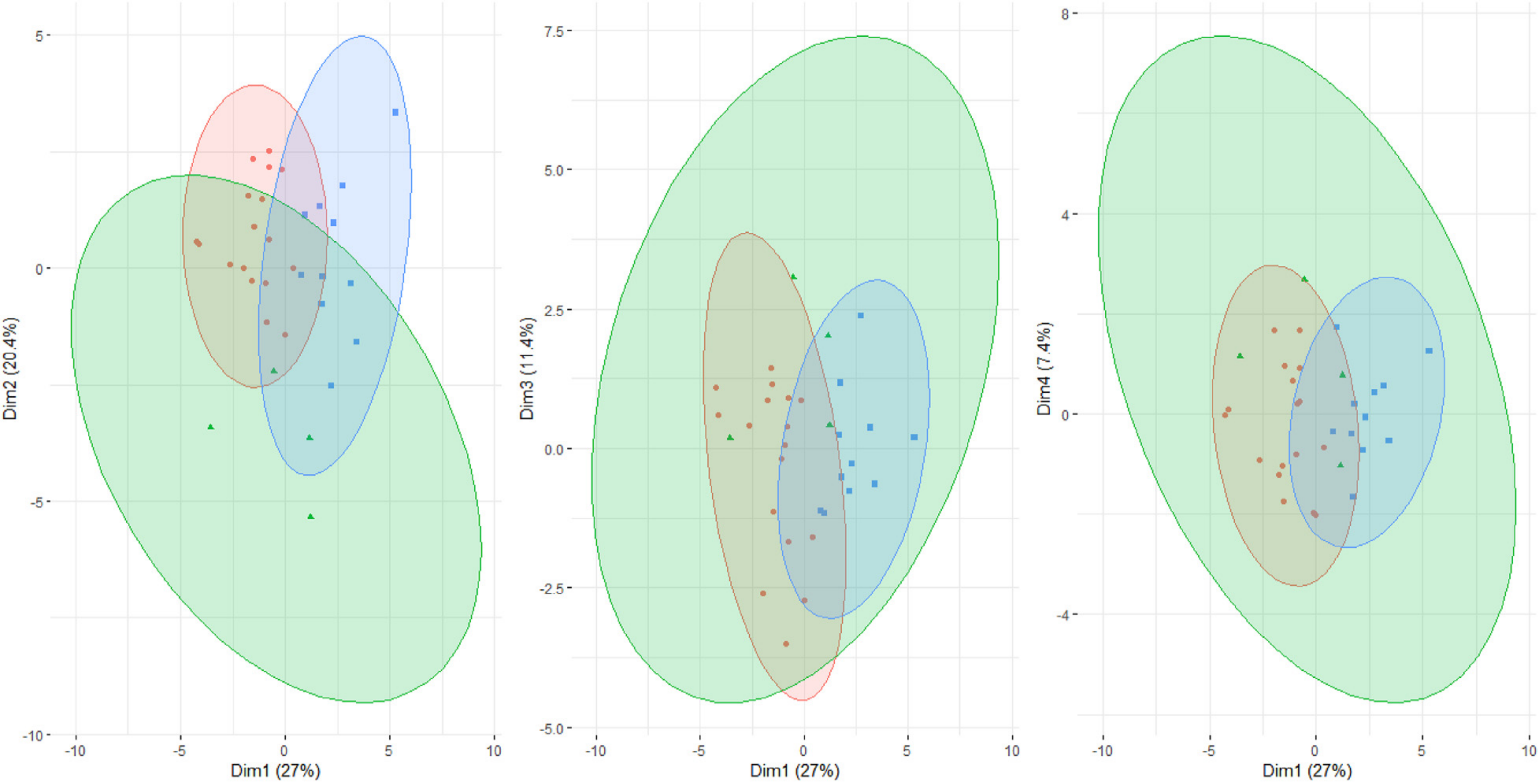
Projection of cultivars in the plane formed by the axes.

The projection of the individuals in the plane formed by 1, 2, 3 and 4 axes showed 3 groups of individuals closely related to the groups of characters previously identified (Figure 7).

### 4.6 Hierarchical ascending classification (HAC)

**Figure 8:**
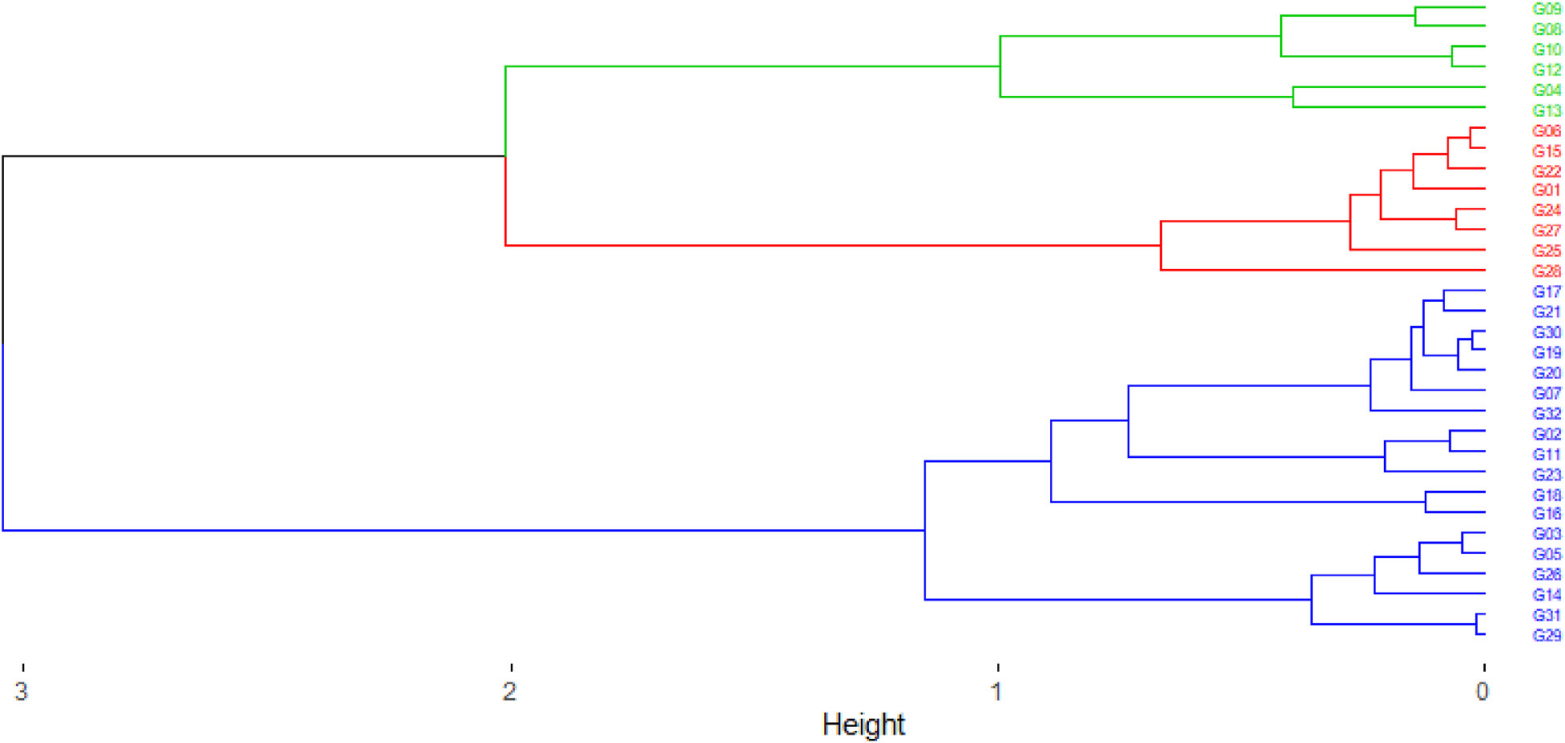
Dendrogram of cultivars based on quantitative traits.

The hierarchical ascending classification was carried out on the basis of the 18 quantitative parameters in order to better appreciate the agro-morphological diversity of maize cultivars. The dendrogram (Figure 8) shows the results of this classification. Three distinct groups of statistically homogeneous cultivars can be distinguished on the dendrogram, confirming the results of the principal component analysis.

#### 4.6.1 Analysis of variance

The results from the HAC were tested by analysis of variance of quantitative traits. The ANOVA model was used as a test for the variables verifying the conditions of normality and homogeneity (Table 12). The result demonstrated that the classes constituted, indicates a very highly significant difference with regards to the different characters except for the average number of grains per row (NmGr) and the weight of thousand grains (PmG).

**Table 12:** Significance of quantitative traits with ANOVA.

	Df	Sum Sq	Mean Sq	F value	Pr (>F)
NrgE	31	296.71	9.57	3.417	6.89e-09 ∗∗∗
Hpla	31	178897	5771	11.159	<2.2e-16∗∗∗
NepP	31	2.16	0.069	2.183	0.0002274 ∗∗∗
LoEp	31	47307	1526	3.735	3.601e-10 ∗∗∗
DiEp	31	2454.8	79.2	3.218	4.242e-08 ∗∗∗
NmGr	31	1634.79	52.74	1.2004	0.2648
PmG	31	24770	799	0.596	0.942
CiCo	31	795.59	25.66	9.542	<2.2e-16∗∗∗
LonF	31	33855	1092	11.345	<2.2e-16∗∗∗
LarF	31	273.6	8.8	2.043	0.0007363∗∗∗
DaFf	31	3065	98.9	12.791	<2.2e-16∗∗∗
DaFm	31	2799.6	90.3	12.66	<2.2e-16∗∗∗
LonP	31	1193.9	38.5	2.694	2.232e-06∗∗∗
DrPa	31	2384.7	76.9	8.462	<2.2e-16 ∗∗∗
NbpP	31	4827.4	155.7	9.858	<2.2e-16∗∗∗
NfEp	31	283.9	9.2	2.019	0.0008905∗∗∗
Hepi	31	169547	5469	20.886	<2.2e-16∗∗∗
CsMa	31	5768.2	186.1	37.002	<2.2e-16∗∗∗

Signif. codes: 0 '∗∗∗' 0.001 '∗∗' 0.01 '∗' 0.05 '.' 0.1 ' ' 1.

#### 4.6.2 Composition of the groups

The characteristics of each group are as follows (Table 13):

**Table 13:** Discriminant variables for each group in the dendrogram.

Characters	Group 1		Group 2		Group 3	
	Mean	Standard deviation	Mean	Standard deviation	Mean	Standard deviation
LonF	68.309	5.291	53.160	9,601	**79.736**	4.624
LarF	8.617	0.739	6.71	1.250	**10.064**	0.496
NmGr	21,330	2,13	23.530625	0.685	**27.758**	1.375
NfEp	5.303	0.361	4.330	1,371	**5.827**	0.349
DiEp	41.792	5.490	45.096875	1.477	**47.837**	1.285
Hepi	68.247	5.851	**95.6125**	15.124	85.654	8.094
DaFf	53.618	1.959	49.660	0.472	**55.909**	1.183
NbpP	11.544	2.241	**16.9375**	3.179	15.009	1.292
DaFm	51.235	1.926	45.661	0.952	**53.364**	1.281
NepP	1.032	0.182	**1.1475**	0.050	1,060	0,251
LonP	28.161	5.900	**32.3875**	2.707	27.962	6.573
LoEp	98.703	18.622	**104.7225**	11.231	93.922	28.833
DrPa	**8.591**	1.002	7.103	2.212	6.363	1.654

The bold numbers represent the highest mean among the 3 distinct groups.

Group 1: This is the largest group. It consists of 18 cultivars (56.25% of the total number) with LonF (68.31 ± 5.29cm), LarF (8,62 ± 0.74cm), NfEp (5.30 ± 0.36), HEpi (68.25 ± 5.85cm), DaFf (53.62 ± 1.96d), NbpP (11.54 ± 2.24), DaFm (51.23 ± 1.92d) and DrPa (8.59 ± 1.002cm). These cultivars therefore had the best vegetative growth. They are intermediate maturing cultivars with the best vegetative, reproductive and ear insertion characters.

Group 2: It is made up of 8 cultivars (25% of the total collection) of which 2 cultivars from the south of Benin and 7 from the north. This group is defined by the variables LonF (79.74 ± 4.62cm), LarF (10.06 ± 0.49cm), NmGr (27.76 ± 1.35), NfEp (5.827 ± 0,35), DiEp (47.84 ± 1.28cm), HEpi (85.65 ± 8.09cm), DaFf (55.91 ± 1.18d), DaFm (53.36 ± 1.28d) and NbpP (15.01 ± 1.29). These are late maturing cultivars with the best vegetative and reproductive traits.

Group 3: Composed of 6 cultivars (i.e. 18.75% of the total number) all collected in southern Benin. This class is characterized by the variables NmGr (23.53 ± 0.68), DiEp (45.10 ± 1.48cm), HEpi (95.61 ± 15.12cm), NbpP (16.94 ± 3.18), NepP (1.15 ± 0.05), LonP (32.39 ± 2.71cm) and LoEp (104.72 ± 11.23cm). This category included early-maturing cultivars with the best growth and the best productivity in terms of ears and grains.

## 5 Discussion

Morphological characterization is one of the important steps in the description and classification of crop germplasm [20, 21, 22]. Data analyses revealed large diversity for all morphological characters from the three experimental sites. This reveals a large inter-cultivar variability. Similarly, the projection of cultivars in the plane formed by axes 1, 2, 3 and 4 of the Principal Component Analysis shows a random distribution of cultivars. This distribution of cultivars in the PCA planes indicates significant agro-morphological variability between cultivars.

Several studies have also revealed a great diversity among maize cultivars with regards to quantitative traits [23, 24, 25]. The use of several varieties by producers seems to be the reasons behind this varietal diversity observed. Indeed, several authors have shown that farmer seed management practices, especially variety exchange between farmers, are at the origin of a significant diversity among crop populations [13, 26, 27, 28]. Moreover, the predominance of poly-varietal cultivation as a field management method of varieties seems to be also responsible for this observed varietal diversity, considering the preferentially allogamous reproduction mode of maize (96%).

The significant differences between cultivars for the different quantitative characters measured confirmed the diversity that exists between these cultivars. It should be noted that the parameters of ear and yield, days to flowering and plant height contributed to the diversity observed between cultivars. These characters allowed to distinguish early and intermediate maize groups, of medium size, from late and the tallest maize groups. Similar results were obtained by [29] using 200 maize accessions grown in central Côte d'Ivoire. Also earliness, plant height, ear insertion height and ear-related traits were used to describe the variability of maize variety populations grown in Cuzalapa, Mexico by [30]. This structuring of morphological diversity shows that in cultivated maize, morphological differentiation is often based on agronomic traits [31]. Phenotypic selection based on perceptible traits (phenology, vegetative, ear) by farmers could explain the contribution of these variables to the structuring of variability. The authors [32] have observed that phenotypic selection based on ear characters contributes to maintaining phenotypic differentiation between maize varieties despite important gene flow. The importance of these quantitative traits in structuring the diversity of maize populations has been highlighted by several authors [30, 33]. The importance of semi-bloom duration in the differentiation of accessions is due to the fact that all the descriptors involved in the distinction are linked to this descriptor [25]. In addition to these characters, the agro-ecological factor also plays an important role in the structuring of morphological diversity.

The percentages of variances expressed by the 4 main axes in principal component analysis for the quantitative variables (26.98–7.39%) indicate the consideration of predominant agromorphological traits for the descriptors considered. A good representativeness of the different axes expresses the existence of a good genotypic and phenotypic organization between the cultivars [34].

Among the 18 quantitative morphological traits, 12 are highly correlated (r > 0.5) with the principal component axes, indicating that the most remarkable phenotypic traits in the field would influence the selection made by the farmers. Indeed, the most interesting cultivars will be those with the best yield parameters.

The analysis of variance of all the parameters used for the constitution of the classes forming the dendrograms showed a significant difference except for NmGr and PmG. The characters are grouped into the classes not according to their origins, but according to their performance in relation to the studied traits, except for some cultivars. On the other hand, the study of [35] revealed the existence of inter-cultivar genetic variability through three groups of cultivars identified according to their geographical origin in Burkina Faso. The local cultivars are practically different from each other for the majority of the traits studied.

The cultivars with the best vegetative and productivity traits revealed by the analyses are those in groups 2 and 3 of the hierarchical ascending classification based on quantitative traits. In addition, the white and yellow corn cultivars widely consumed and appreciated by users can be found among the cultivars of these two groups.

In sum, quantitative parameters, although strongly influenced by environmental conditions, should be considered by curators in genetic diversity studies because they are essential in the farming environment and significantly influence phenotypic selection criteria.

## 6 Conclusion

The study of the agro-morphological diversity of maize cultivars collected from the center, south and north of Benin clearly shows that the cultivars present a variation for all the traits studied, particularly those related to phenology, morphology and yield. This genetic variability observed between cultivars constitutes an asset for varietal selection program. It also shows that the phenotypic variability of cultivars is more related to their agronomic and morphological traits than to their origin. The diversity analysis structured three groups by hierarchical ascending classification. Each group thus constitutes a stream of traits of interest for maize improvement in the study area. The differences observed between the clusters from the hierarchical clustering indicate that cluster 1 is made up of intermediate maturing cultivars of large size with inserted cobs at a great height. In contrast, clusters 2 and 3 contain cultivars with the best ear and kernel characters and can be used as a source of breeding stock for a corn yield improvement program. These results are an intermediate step in the variety improvement process. It is important to combine morphological traits with molecular techniques to better characterize cultivars within the different groups.

## Declarations

### Author contribution statement

Vincent Ezin: Conceived and designed the experiments; Analyzed and interpreted the data; Contributed reagents, materials, analysis tools or data; Wrote the paper.

Chabrolle M. I. Kpanougo: Conceived and designed the experiments; Performed the experiments; Analyzed and interpreted the data; Wrote the paper.

Adam Ahanchede: Conceived and designed the experiments; Wrote the paper.

### Funding statement

This research did not receive any specific grant from funding agencies in the public, commercial, or not-for-profit sectors.

### Data availability statement

Data included in article/supp. material/referenced in article.

### Declaration of interests statement

The authors declare no conflict of interest.

### Additional information

No additional information is available for this paper.
